# Cybersecurity Risk Analysis of Electric Vehicles Charging Stations

**DOI:** 10.3390/s23156716

**Published:** 2023-07-27

**Authors:** Safa Hamdare, Omprakash Kaiwartya, Mohammad Aljaidi, Manish Jugran, Yue Cao, Sushil Kumar, Mufti Mahmud, David Brown, Jaime Lloret

**Affiliations:** 1 Department of Computer Science, Nottingham Trent University, Nottingham NG11 8NS, UK; safa.hamdare2021@my.ntu.ac.uk (S.H.); mufti.mahmud@ntu.ac.uk (M.M.); david.brown@ntu.ac.uk (D.B.); 2 Computer Science Department, Faculty of Information Technology, Zarqa University, Zarqa 13110, Jordan; mjaidi@zu.edu.jo; 3 JMVL Limited, Jenkins Avenue, Bricket Wood, St Albans AL2 3SB, UK; manish.jugran@jmvl.co.uk; 4 School of Cyber Science and Engineering, Wuhan University, Wuhan 430072, China; yue.cao@whu.edu.cn; 5 School of Computer and Systems Sciences, Jawaharlal Nehru University, New Delhi 110067, India; skdohare@mail.jnu.ac.in; 6 Instituto de Investigación para la Gestión Integrada de Zonas Costeras, Universitat Politécnica de Valencia, Camino Vera s/n, 46022 Valencia, Spain; jlloret@dcom.upv.es

**Keywords:** EV charging, cybersecurity, energy network

## Abstract

The increasing availability of Electric Vehicles (EVs) is driving a shift away from traditional gasoline-powered vehicles. Subsequently, the demand for Electric Vehicle Charging Systems (EVCS) is rising, leading to the significant growth of EVCS as public and private charging infrastructure. The cybersecurity-related risks in EVCS have significantly increased due to the growing network of EVCS. In this context, this paper presents a cybersecurity risk analysis of the network of EVCS. Firstly, the recent advancements in the EVCS network, recent EV adaptation trends, and charging use cases are described as a background of the research area. Secondly, cybersecurity aspects in EVCS have been presented considering infrastructure and protocol-centric vulnerabilities with possible cyber-attack scenarios. Thirdly, threats in EVCS have been validated with real-time data-centric analysis of EV charging sessions. The paper also highlights potential open research issues in EV cyber research as new knowledge for domain researchers and practitioners.

## 1. Introduction

**Figure 1:**
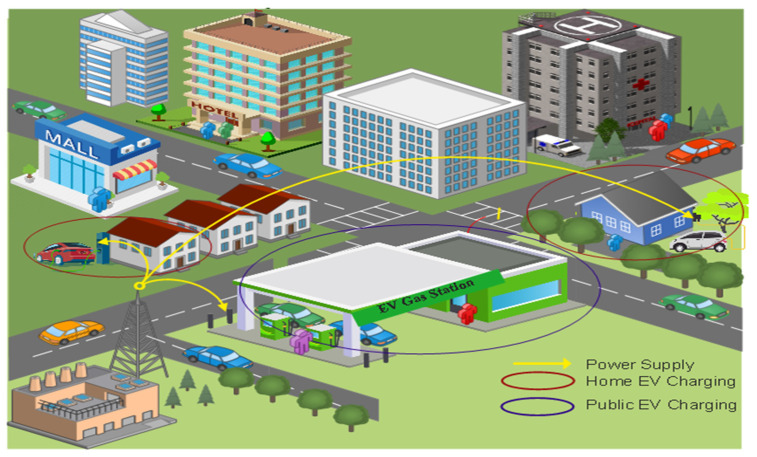
EVs Charging Infrastructure.

Energy management and transportation systems that use artificial intelligence have become more significant in modern cities as they develop major urban infrastructures. As a result, Electric Vehicles (EVs) will be more commonly used as part of private and public transportation fleets in the future (Figure 1). Government has backed various initiatives to promote the usage of EVs, concentrating on the contribution to a wide range of future green transportation policy goals [1]. EV usage enhances air quality, reduces noise pollution, and cuts carbon emissions by eliminating road traffic pollution. As per the Accelerating to Zero (A2Z) mandate to reduce carbon emissions, several governments worldwide have taken steps to reduce fossil-fuel-driven vehicles. The UK government has also signed up to work towards bringing in new cars and vans with zero emissions. To help the UK government reach its 2050 “Road to Zero greenhouse gas emission” goal, EVs play a significant role. As of 2030, the sale of gasoline and diesel automobiles is proposed to be banned in the UK, with the sale of hybrid vehicles to follow in 2035 [2]. This sale analysis follows the suggestion of the Committee on Climate Change [3] that the EVs market is set to reach 100% of all vehicle sales by 2035 to achieve the net zero ambition of the UK.

EVs charging ecosystem is a connected system paradigm at the core of the smart grid, consisting of a complex cyber-physical system compromising linked hardware parts, software elements, and communication protocols. Power from the grid is transferred to EVs using EVCS. The EVCS is a self-contained and Internet-of-Things-enabled infrastructure that operates on its proprietary firmware. The public EVCS is controlled by a cloud server which allows users to be guided in the direction of available EVCS, set up and manages charge sessions, and keep track of consumption statistics. Users of public EVCS communicate with the charging management system through the Internet. Usually, users schedule charging sessions, set the charging rate, begin, and end charging, and check on the status of their EVs using these services. The power infrastructure must be functional and connected to charge an EV. Because EVCS is connected into the grid and takes the necessary power from it, it poses a significant threat to the reliability and safety of the power supply. All data exchanged among the user application, EV, and EVCS must be secured to guarantee the safety and reliability of the ecosystem. Equipment manufacturers, national governments, and EVCS operators have their preferred protocols for enabling cybersecurity. Inconsistencies caused by a lack of standardization of protocols lead to severe cybersecurity problems [4].

However, EV charging station cyberattacks have yet to be taken seriously on a wider industry scale or at the government policy level. However, the smart application created for home EV charging was found to have security issues by Kaspersky Lab [5]. The charging process for EVs might be disrupted if an attacker would gain access to the charging equipment via the Wi-Fi connection. Schneider EVs link chargers were also found to have security issues [6], allowing remote attackers to deceive hard-coded passwords, insert malware, and deactivate the charger with this vulnerability.

In this context, this paper presents a critical analysis of EVs charging ecosystem considering potential cyber threats, loopholes present in the charging network, major charging parameters, and communication protocols. The main contributions of the paper are the following:

(1) The potential cyber vulnerabilities in EVs charging ecosystem are identified, which could be linked to potential threats.
(2) It exploits some of the coexisting cybersecurity attacks and their possible use in EV charging networks.
(3) The literature on security issues in EV charging is explored, thoroughly focusing on cyber-attack points and vulnerable communication protocols.
(4) A critical cyber threat analysis of EV charging sessions is done based on real-time charging data and parameters. This analysis can be linked to cyber risks in EV charging networks.
(5) Open cyber research issues have been precisely highlighted in the EV charging ecosystem as new knowledge for domain researchers.

The rest of the article is organized into the following sections. Section 2 covers the background on recent advancements in EVCS. Section 3 presents cybersecurity needs for EVCS. Cybersecurity threat analysis for EVCS is discussed with real data in Section 4. Section 5 highlights potential open research issues in EVCS, followed by a conclusion presented in Section 6.

## 2. Background

### 2.1. Advancement in EV Ecosystem

**Figure 2:**
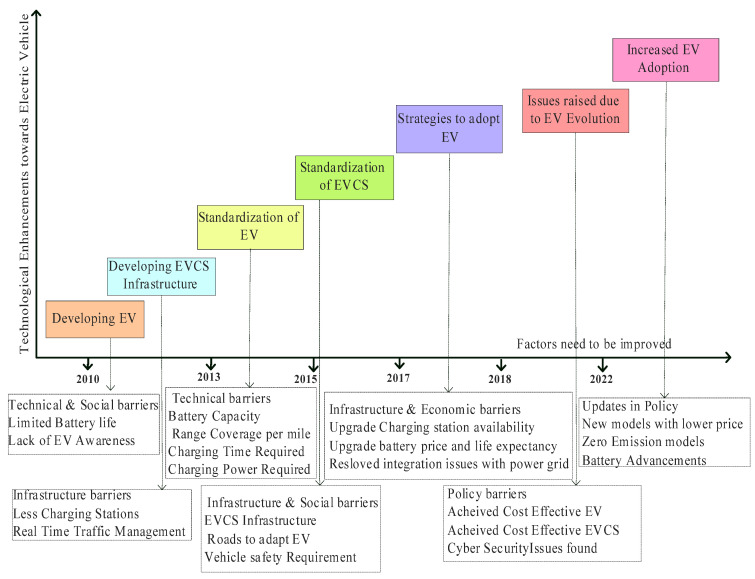
Roadmap of Technological Enhancements in EVs.

The use of EVs has yet to be common in the car industry. Some people are worried about running out of power while driving because there are not enough places to recharge their vehicles. That is why the government offers money-saving incentives like tax breaks and rebates to encourage people to buy EVs. Advancements in EVs are highlighted in (Figure 2), explaining various aspects of EV technology and infrastructure from 2010 onwards, with significant years highlighted.

The decreasing cost of EVs can be attributed to the advancements in technology utilized in their production and the scaling up of their manufacturing processes. This advancement has helped change people’s opinions about EVs. The technological revolution in battery development has been identified as a potential catalyst for promoting Electric Vehicle (EV) adoption [7]. The cost of batteries has also gone down, which has made up about a quarter of the total cost of an EV. EVs have problems, like needing more charging stations, which is challenging for people who own them [8]. Efforts to address this challenge necessitate the establishment of an efficient and intelligent network of charging stations. Leveraging algorithms to optimize charging station allocation for individual users could significantly reduce wait times and boost productivity.

Additionally, promoting home charging can aid in prolonging battery life while also contributing to grid stability [8]. This study compares EVs’ extant production and testing with new designs currently in the prototype phase [9]. Researchers have examined how far an EV can go on one charge, how big the battery is, how powerful the charger is, and how long it takes to charge the vehicle. They also discussed the charging stations’ and vehicles’ specifications and how this affects the power grid. Another study also looked at the safety requirements for the vehicles and the roads they drive on [10]. The recommendation is to build more charging stations quickly to cope with the growing number of EVs on the road. The study found that different chargers are available for EVs with different power levels and interfaces. The common barriers to EV station advancement include issues such as cost, regulatory permissions, and theft. Recommendations for overcoming these hurdles were made, including increasing the attractiveness of EV ownership by making EV charging stations accessible to the public. Proper placement of charging stations to ensure widespread EV adoption is critical to mitigate some of the inherent risks associated with this technology [11]. Some of these issues include factors such as battery price, battery life expectancy, the availability of charging stations, integration issues with the smart grid, range, and coverage. This research was carried out from three perspectives: charging stations, batteries, and vehicle types. In recent work [12], the difficulties that have arisen throughout the evolution of EVs in recent years are examined. The total expense of owning a battery-operated EV has decreased significantly due to lower installed battery prices, and this trend is expected to continue. The efficient and cost-effective deployment of charging infrastructure is significantly more critical for the long-term growth of EV ownership.

### 2.2. EV Adaption Trend

The main factor responsible for EV adoption is the types of charging used. The three main types of electric vehicle (EV) charging are Level 1, Level 2, and DC fast charging (DCFC), and they have different impacts on the EV charging experience.

- Level 1 charging uses a standard household outlet (120 volts) and provides a slow charge rate of around 2–5 miles of range per hour of charging [13]. This type of charging is best suited for overnight charging at home and is convenient for EV owners with low daily driving needs.
- Level 2 charging uses a dedicated charging station that operates on 240 volts and provides a faster charge rate of 10–60 miles of range per hour of charging, depending on the vehicle and the charging station’s power output [13]. Level 2 charging is commonly found in public locations such as shopping centers, workplaces, and public parking facilities, and it is suitable for daily charging needs.
- DC fast charging (DCFC) is the fastest type of EV charging. It can provide up to 80% of a vehicle’s battery capacity in around 30 min, depending on the vehicle and the charging station’s power output [14]. DCFC stations are typically located along highways and major travel routes, making them ideal for long-distance travel.

**Figure 3:**
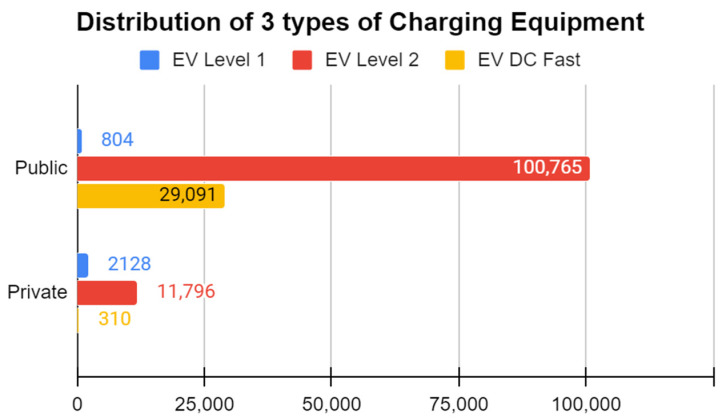
Factors influencing adoption of EVs.

The EV charging industry trend has been toward expanding Level 2 and DCFC charging infrastructure. This trend is because Level 2 charging provides a faster and more convenient charging experience than Level 1 charging, and DCFC stations are essential for long-distance travel and reducing range anxiety. This trend can be explained by the analysis shown in (Figure 3).

In recent years, many public charging stations and automakers have invested heavily in DCFC infrastructure to make long-distance travel in EVs more practical and convenient. As a result, the number of DCFC stations has grown significantly, making it easier for EV owners to travel longer distances without worrying about running out of charge. The impact of Level 1, Level 2, and DCFC charging on the EV charging experience varies based on charging speed, charging location, and the driver’s charging needs. The EV charging industry trend has been toward expanding Level 2 and DCFC infrastructure, as these types of charging provide a faster and more convenient charging experience for EV owners.

### 2.3. EV Charging Use Case

An EVs charger open to the public is called a “public charging station”. A charging station designed for residential use is typically installed permanently at one’s home, with the user only charged for power consumed. Due to an increase in the use of EVs worldwide, more and more private and public charging stations are being built. Table 1 summarizes the pros and cons of the presented EVCS use cases.

**Figure 4:**
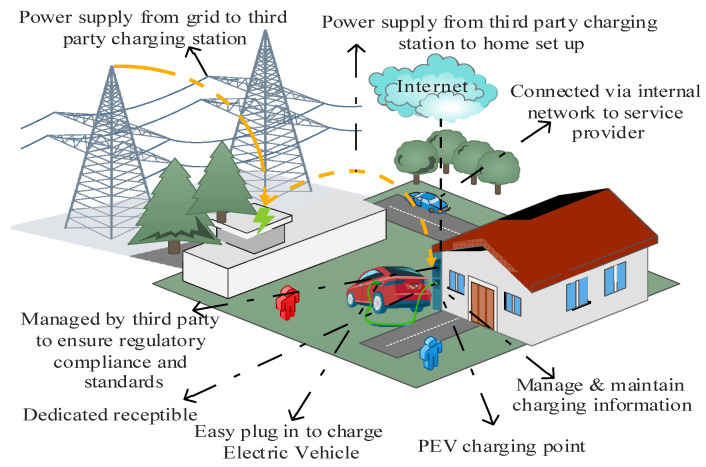
EV Home Charging use case scenario.

As illustrated in (Figure 4), the private charging use case describes the home charging use case where EVs can be recharged at home. It is safer and less hazardous to charge an EV at home since the EV is connected to an established network, making it more secure. Charging an EV at home might have some positive effects as well as limitations, as follows:

(1) More convenient: Installing a home charging station presents benefits in terms of saved time and reduced reliance on gasoline. Charging an electric vehicle at home can eliminate waiting in parking areas for charging opportunities.
(2) Increased savings: According to U.S. Department of Energy estimates, charging an EV with a 33 kWh capacity at 0.13 cents per kWh costs only $0.04 per mile [15]. Home charging saves money by avoiding high public charging rates. Residential EV charging stations pay for themselves via cost savings.
(3) Greater home value: EVCSs could increase property value and save time/costs. Homebuyers with EVs seek residences with pre-installed EV charging, boosting demand. This setup could lead to faster sales at higher prices, recouping the initial EVCS investment.
(4) Less wear and tear and safe: Fewer people use it, which reduces wear and tear and repair costs. We know who uses the chargers despite not being connected to any external network.
(5) Longer charging time: Many owners wish to keep the Level 1 charger with their EVs. These chargers charge slower than public ones. A Level 2 charger helps speed up charging. Level 2 chargers are faster than Level 1 chargers, charging batteries up to 30–44 miles per hour [15].
(6) Higher Upfront Cost: If they do not have the money to pay for their own, they will have to rely on public stations. In contrast, individuals with the money to do so will gain long-term savings and increased property value by charging at home.

**Figure 5:**
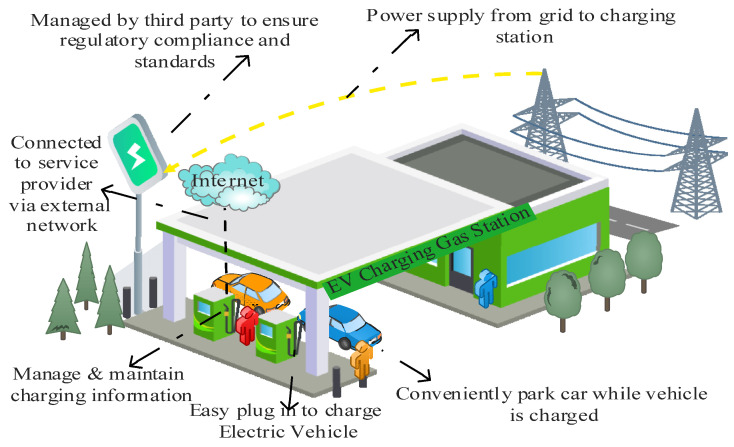
EV Public Charging use case scenario.

The public charging use case illustrated in (Figure 5) presents the second use case in which EVs can be charged in public charging stations. This use case is more vulnerable to assault since it is connected to an external network and can be readily altered.

(1) Using public EV charging instead of home infrastructure has the following advantages and some associated challenges:
(2) Less investment cost: Public EV charging stations do not require substantial initial expenditure. Since the company paid for and built the station, individuals only need to pay for the electricity they use for charging their EVs.
(3) Economical for the public: Public EVCSs may offer faster charging than most homes can afford. Some public chargers are ideal for needing to charge a vehicle urgently during a journey.
(4) Longer public waiting time: When a person arrives at a charging station, they may have to wait a long time. Thus, home charging can be preferred over public charging to minimize long delays.
(5) Inconvenient searching for EVCSs: Finding one may be difficult if someone is unfamiliar with their neighborhood’s charging stations. Even if a person has discovered all the public charging stations on their normal route, having an at-home charger is a smart choice.
(6) Potential damage to battery: Utilizing Level 3 fast chargers may cause an EV’s battery to deteriorate more quickly than with Level 1 or Level 2 chargers [15]. For those who want to increase their battery life, it is advisable to use rapid charging stations rarely and home chargers frequently.

### 2.4. EV Charging Usage Pattern

Home charging is the most convenient and cost-effective way to charge an EV. Many EV owners install a Level 2 charging station at home, allowing them to charge their vehicle overnight while sleeping. This way, they can wake up to a fully charged vehicle each morning and start their day without worrying about finding a charging station. However, in some cases, drivers may need access to home charging due to a lack of dedicated parking or the inability to install a charging station. In these cases, public charging infrastructure becomes essential for EV adoption. The U.S. public and private EV charging infrastructure graph on the alternative fuels data center website visually represents the growth of electric vehicle charging infrastructure in the United States over time [16].

**Figure 6:**
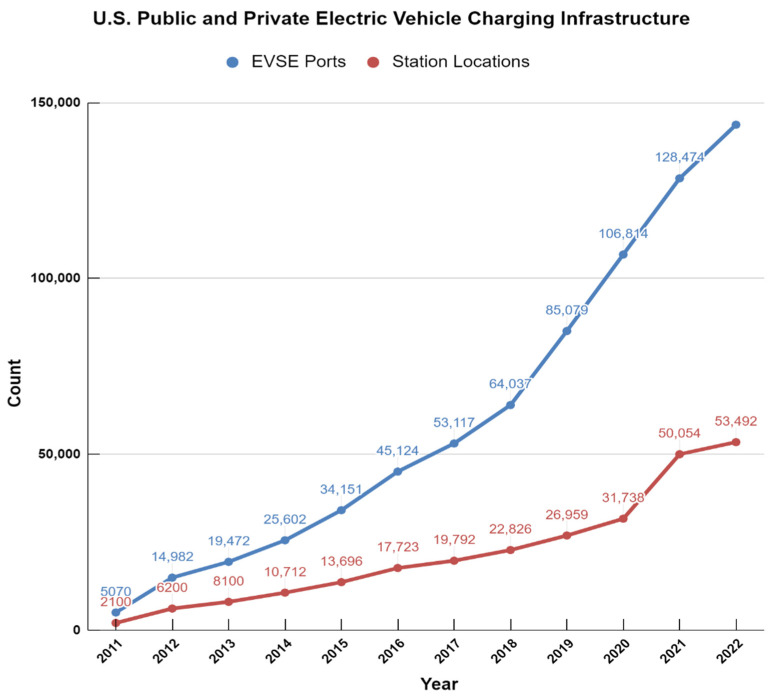
The Trend of U.S. Public and Private EV Charging Infrastructure [14].

The graph (Figure 6) shows two lines, one representing the number of public electric vehicle charging stations in the United States and the other representing the number of private electric vehicle charging stations. The data in the graph cover the period from 2011 to 2021. The trend in the graph shows a steady increase in the number of EVCS in the United States over the years. Public charging stations have grown significantly since 2011, from just over 1000 to over 40,000 stations as of 2021. Similarly, private charging stations increased from just over 600 stations in 2011 to over 10,000 in 2021.

The growth in EV charging infrastructure can be attributed to several factors, including government incentives and policies that encourage the adoption of EVs, advancements in technology that have made EVs more affordable and practical for consumers, and the increasing demand for sustainable transportation options. As the number of EVs on the road continues to grow, the need for charging infrastructure will also continue to increase. The trend in the graph shows that the United States is making significant progress in expanding its EV charging infrastructures, which is essential for promoting the widespread adoption of EVs and reducing carbon emissions in the transportation sector.

Public charging stations are often located in areas where drivers spend time, such as workplaces, shopping centers, and public parking facilities. This convenience makes it easy for drivers to top off their vehicle’s charge during the day while running errands or working. Additionally, some drivers may need to use public charging infrastructure for long-distance travel or to supplement their home charging. For example, drivers may need to use fast charging stations located on highways for quick charging during long road trips. In short, while home charging is often considered the most convenient and cost-effective option for electric vehicle owners, public charging infrastructure is essential for EV adoption, particularly for drivers who do not have access to home charging or need to supplement their charging needs for long-distance travel.

## 3. Cyber Vulnerability in EVCS

### 3.1. Infrastructure Centric Vulnerability in EVCS

**Figure 7:**
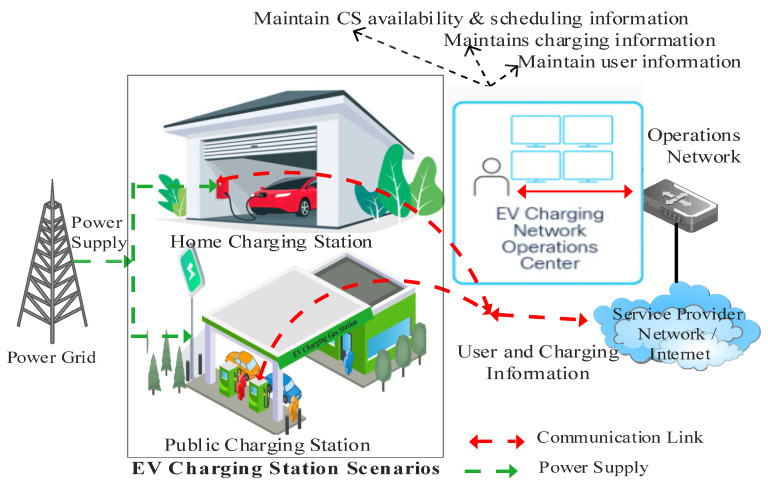
Communication in EVCS Infrastructure.

EVCS infrastructure comprises a power grid, charging station, service provider, and EVs user connected. There is communication among them in the network to maintain data related to charging (Figure 7).

**Figure 8:**
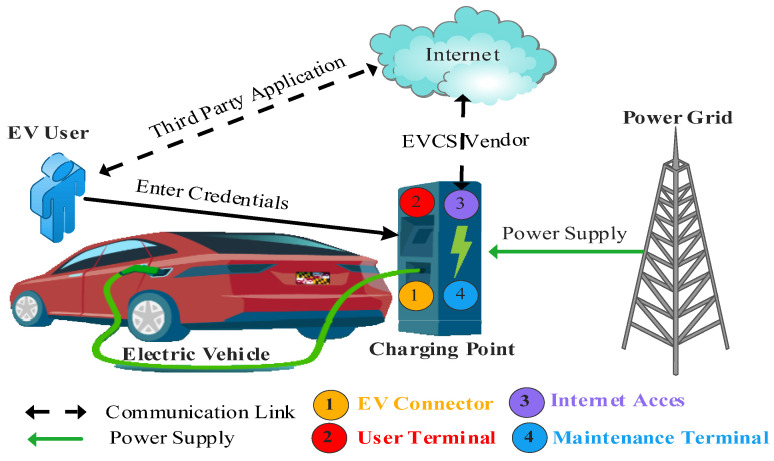
EVCSs with Vulnerable points.

The service provider relates to the operator network to maintain information on energy and time required for specific EVs. The service provider also relates to charging stations to check their availability so they can schedule EVs visits accordingly and connect to EVs for user information related to payment. Researchers have identified vulnerabilities in EVCS devices and their communications among networks, including the cloud services involved. EVCSs security evaluations and vulnerabilities are described by interface type use case [17]. (Figure 8) depicts the four probable entry points used by attackers to compromise EVCSs. Potential security vulnerabilities can arise through various ports of entry in electric vehicle (EV) charging systems, including EV connectors, user terminals, Internet connections, and maintenance terminals. These ports allow attackers to exploit weaknesses and compromise the security of the EV charging infrastructure.

1. EV connectors: EV connectors serve as potential targets for attackers due to their communication protocols and connectivity capabilities. Attackers may exploit these vulnerabilities to introduce malware or manipulate charging settings, gaining unauthorized access to the EVCS. This exploitation can have severe security implications, as malicious actors’ unauthorized access to the EVCS opens the door for further compromise and control. Moreover, side-channel threats pose a significant concern during the charging process.

Attackers may leverage these vulnerabilities to gather sensitive information or indirectly manipulate the EVCS. This attempt compromises the privacy and integrity of the charging system and creates potential risks for the connected vehicles and their owners. Robust security measures should be implemented to ensure the security of EV connectors. These security measures include rigorous testing and validation of communication protocols, implementation of secure coding practices, and continuous monitoring for any signs of malicious activity.

2. User terminals: Public EV charging stations commonly rely on authentication methods such as RFID, NFC, or credit card chips/swipes to connect charging sessions and user accounts, facilitating billing and tracking. However, the security of these authentication systems is crucial, as any compromise could lead to significant consequences for both users and the charging infrastructure.

If attackers manage to compromise these authentication systems, they gain the ability to carry out various malicious activities. They can deactivate charging sessions, causing inconvenience and potential disruptions for EV owners. Furthermore, attackers can manipulate pricing mechanisms, leading to financial losses for users or the charging station operator. Unauthorized modifications to the equipment could introduce safety risks, impacting not only the charging infrastructure but also the vehicles being charged. Implementing strong encryption and secure communication protocols, regularly updating and patching authentication systems, and conducting thorough vulnerability assessments are essential to mitigate the risks of compromise.

3. Internet connections: Integrating Internet connections in modern EVCSs brings convenience and advanced services operators, or EVCS providers offer. Nevertheless, it is essential to acknowledge the associated security risks that arise from this connectivity. Breaching the EVCSs compromises the charging infrastructure and allows attackers to exploit the system as an access point for launching broader attacks on critical infrastructure.

By infiltrating the EVCSs via an Internet connection, attackers can gain unauthorized access to the connected network, extending beyond the charging infrastructure. This entry point could enable them to target and manipulate the critical components of the power grid or transportation network. The consequences of such attacks could be severe, leading to disruptions in power supply and transportation systems or even compromising public safety. Robust security measures should be implemented, including strong access controls, encryption protocols, intrusion detection systems, and regular security updates.

4. Maintenance terminals: EVCSs typically comprise multiple circuit boards communicating through the Ethernet or serial or analog interfaces [18]. One significant concern is the lack of encryption in module communications, which leaves these communications vulnerable to eavesdropping or tampering by unauthorized individuals. Additionally, the presence of physical ports intended for maintenance purposes can inadvertently create potential access points for attackers if overlooked during production or not properly secured.

Exploiting these overlooked openings, attackers could gain unauthorized access to the EVCSs, compromising the integrity and security of the entire system [19]. They may monitor sensitive information exchanged between the maintenance terminal and the EVCSs. Moreover, malicious actors could disrupt the operation of the EVCSs, leading to service disruptions, financial losses, or even safety hazards. Implementing robust encryption protocols for module communications, employing secure authentication mechanisms, and conducting regular security audits to address these security concerns is essential. Physical security measures such as securing physical ports and accessing controls should also be implemented to prevent unauthorized tampering or access to maintenance terminals.

Addressing these security aspects is crucial to ensure the integrity and safety of EV charging systems. Robust security measures, including encryption, authentication mechanisms, and regular security audits, should be implemented to mitigate the risks associated with these ports of entry.

### 3.2. Difference in Threats of EV Network and Classical Network

EVs bring forth unique security challenges that differentiate them from classical network security. The following point discusses the specific security aspects of EV networks and highlights the key differences compared to traditional network environments:

1. Unique Communication Protocols: EVs rely on specialized communication protocols for vehicle-to-vehicle (V2V) or vehicle-to-infrastructure (V2I) communication. These protocols introduce novel vulnerabilities and attack vectors distinct from those in traditional network security. Implementing these protocols requires careful consideration to ensure secure and reliable communication within the EV ecosystem.
2. Physical and Cyber Integration: Integrating physical components (such as batteries and charging stations) with cyber systems (including in-vehicle software and charging infrastructure networks) creates a complex interplay between the physical and digital realms. Security breaches in either component can have far-reaching consequences, impacting the overall safety and functionality of the EV system. Protecting against such threats requires a comprehensive approach encompassing both physical and cybersecurity measures.
3. Battery Security: The security of EV batteries is paramount, as they serve as the primary energy source. Unauthorized access or manipulating battery systems can lead to severe safety risks, such as fire incidents or compromised vehicle performance. Robust security measures must be in place to safeguard EV batteries from unauthorized access, tampering, or malicious attacks.
4. Charging Infrastructure: EVs heavily rely on charging infrastructure, which presents its own set of security concerns. Securing charging stations is essential to prevent unauthorized access to billing systems, ensure the integrity of transactions, and protect against potential attacks on the power grid. The interconnected nature of charging infrastructure necessitates robust security mechanisms to maintain the reliability and trustworthiness of the charging process.
5. Privacy Concerns: EVs gather and process sensitive driving behavior, location, and energy consumption data. Protecting the privacy of these data poses unique challenges beyond classical network security considerations. Safeguarding personal information from unauthorized access and ensuring responsible data handling practices are vital to address privacy concerns in the EV ecosystem.

EV network security stands apart from classical network security due to electric vehicles’ unique characteristics and requirements. The utilization of specialized communication protocols, the integration of physical and cyber components, battery security, the protection of charging infrastructure, and the handling of privacy concerns necessitate tailored security approaches. By acknowledging and addressing these distinctive security aspects, we can build a robust and resilient EV network infrastructure that ensures EV users’ safety, privacy, security, and the overall ecosystem.

### 3.3. Protocol-Centric Vulnerabilities in EVCS

Data exchange between the EVs and the EVCSs is outlined in the International Electrotechnical Commission’s (IEC) and International Organization for Standardization (ISO) standards. Unfortunately, there are flaws and security holes in this mode of communication. Although initially developed for substation control systems, IEC protocols are now a part of the EVs infrastructure. Protocols such as IEC 61850-90-8 [20] and IEC 61851-1 [21] describe several features of EV charging, but we will only be looking at two for now. The needs of smart charging are met by IEC 61850-90-8, which has considered other standardization initiatives from the start. Some fundamental features for EV charging, such as user identification, were found to be lacking for this protocol [4] and instead assigned to others, such as the Open Charge Point Protocol (OCPP) [22] or other IEC protocols.

As defined in ISO/IEC 15118 [23], which allows for digital communication in both directions, this international standard supplement the existing IEC 61851-1 [4]. ISO 15118 is neither privacy-friendly nor demand response-compliant, except for a clause specifying that sensitive data should only be disclosed to those who need to know. Previous research raised privacy concerns, and a real-world attack was attempted [24]. Some flaws in the protocol or incorrect use of existing security mechanisms have been brought to light [25] and are addressed as follows:

(1) The Signal-Level Attenuation Characterization protocol supports mutual authentication and encrypted communication, allowing it to function securely.
(2) Even though this protocol is Transport Layer Security compliant, encryption is turned off after an external authority verifies the charging session as safe.
(3) ISO 15118 also facilitates the establishment of public key infrastructure.

Nevertheless, these safeguards are optional; most manufacturers overlook them to save money and effort. This negligence has left plain-text communications open to assault. Real-time remote control of the EVCSs is made possible by OCPP. This feature facilitates the exchange of data and energy between the EVCSs, the EVs management system, the EVs, and the grid.

**Figure 9:**
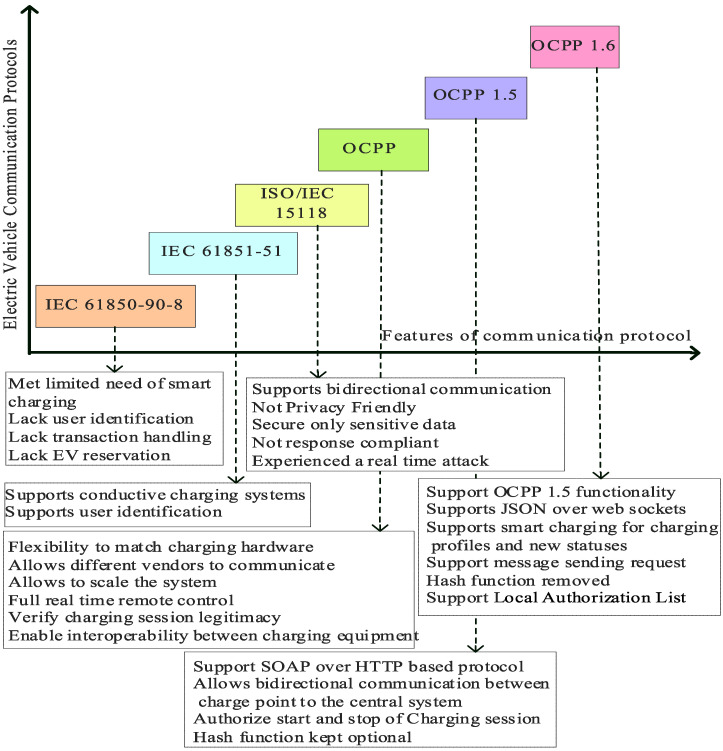
Roadmap of EVs Communication protocol.

The most widely adopted charging protocol today is OCPP, known for its standardization. As well as initiating and terminating charging sessions and processing bills, OCPP allows online changes to be made to the charging settings. OCPP supports smart charging by regulating session timing, charging rate, and charging time. OCPP uses many communication protocols to control EV charging but only utilizes HTTP for management. Despite the releases of OCPP 2.0 and 2.0.1 in 2018 and 2020, respectively, OCPP 1.5 and 1.6 are still widely used [19]. Over Web Sockets, OCPP 1.6 supports various communication frameworks, including SOAP/XML and JavaScript Object Notation. Most manufacturers and operators have disregarded OCPP’s optional TLS layer for a secure connection to save costs. Extra security was available through an optional hash function in OCPP 1.5. However, OCPP 1.6 removed this option instead of requiring it in further releases. EVs communication protocol road map is presented in (Figure 9).

Verifying a charging session’s legitimacy with a billing system is the primary focus of OCPP’s security measures [26]. As a result, attackers may easily hijack the transmission and take control of EV charging since it is conducted in plain text and encryption is not widely used. Even if an attacker cannot decipher the transmitted data, they may still be able to interrupt an EV’s charging process by intercepting and replaying communications, such as those that initiate and end charging sessions. The local authorization list features available in OCPP versions 1.5 and 1.6 ensures that an EVCS can continue to serve its customers during a network outage [27].

The series of IEEE 1547 [28] tackles many of the technical integration difficulties for a mature smart grid, including high penetration of distributed generators, grid support, and load control. These are some of the issues that are addressed [29]. The smart grid interoperability standard requires more of a layered security approach. Cybersecurity solutions supplier C2A Security [30] has introduced a new cybersecurity management system called EVSec, which automates EV’s infrastructure security. By providing an automated and centralized solution, EVSec can meet the cybersecurity demands of the entire electric vehicle infrastructure.

### 3.4. Cybersecurity Attacks in EVs

**Figure 10:**
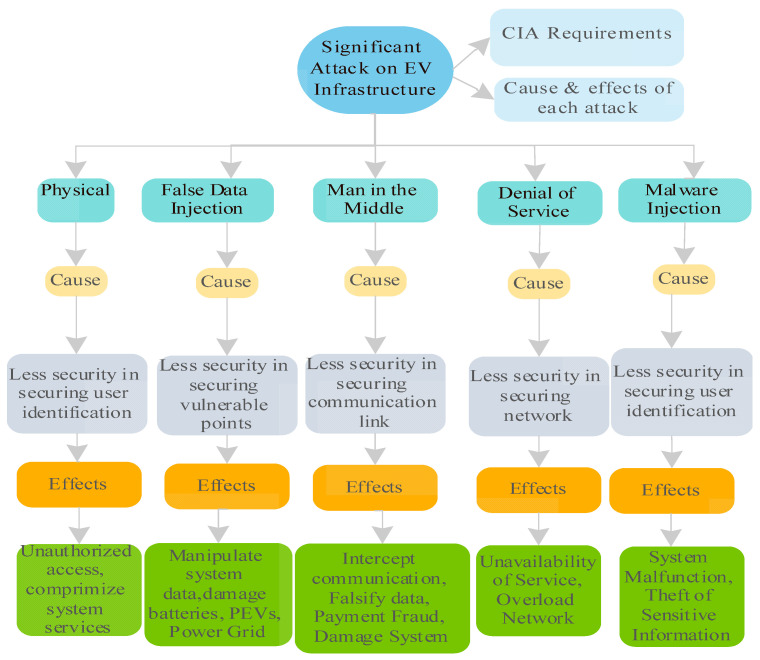
Significant attacks on EVCSs.

Power grid, charging stations, service providers, and EVs users are all linked to Smart Charging Management Systems (SCMSs) and Electric Vehicle Supply Equipment (EVSE). As a result, the power grid might be affected, and Plug-in Electric Vehicle (PEV) batteries can be easily damaged. An EVSE system’s accessibility and power consumption might be used to interrupt a building’s power supply to a specified region. The disruption would be more severe if the hacker also placed persistent malware in the EVSE, which then propagated to the SCMS and the power grid. This means that SCMS and its interconnected system will fall short of meeting the CIA requirements. The following is a breakdown of the most significant attacks that could be carried out on SCMS and its network, summarized in (Figure 10).

(1) False Data Injection: False data injection in an EVCS involves an attacker gaining unauthorized access to the communication channels within the system. By exploiting vulnerabilities in the communication protocols, the attacker intercepts data transmission between the EVSEs, smart measuring equipment, and the SCMS. They then manipulate or inject false data related to PEV charging and discharging, such as altering charging rates or battery status [19]. This exploitation can deceive the SCMS, leading to incorrect decision-making and potentially harmful consequences. The impact includes overcharging batteries, compromised vehicle performance, and disruptions to grid stability. Preventive measures such as secure communication protocols, encryption, and authentication mechanisms are necessary to mitigate this attack and ensure the integrity of charging data in the EVCS.
(2) Man-in-the-Middle: In an EVCS, a Man-in-the-Middle (MITM) attack occurs when an unauthorized attacker inserts themselves between the communication channels of the system. The attacker intercepts and manipulates the data transmission between the Electric Vehicle Supply Equipment (EVSE), the Plug-in Electric Vehicles (PEVs), and the Smart Charging Management System (SCMS) [27]. By gaining access to the communication flow, the attacker can alter, discard, or misrepresent the data exchanged, leading to various malicious outcomes [4]. For instance, the attacker can tamper with charging requests, leading to overcharging or over-discharging PEV batteries, potentially damaging or reducing their range [4]. Additionally, the attacker can exploit this position to breach privacy by accessing sensitive information exchanged between the PEVs and the SCMS. To mitigate MITM attacks in the EVCS, robust encryption, authentication mechanisms, and secure communication protocols should be implemented to ensure the integrity and confidentiality of the data transmission [31].
(3) Denial of Service: In an EVCS, a Denial-of-Service (DoS) attack aims to disrupt the system’s normal functioning by overwhelming it with excessive traffic or requests [32]. In this attack, an adversary targets the SCMS or associated components to overload the network, making it unable to provide services to legitimate users. The attacker may flood the system with high charging requests, exhaust system resources, or exploit vulnerabilities to crash the SCMS. As a result, the system may become unresponsive, preventing PEVs from accessing the charging services. Such an attack can have severe consequences, particularly for critical emergency vehicles that require charging, potentially compromising their availability and hindering emergency response efforts [33]. To counter DoS attacks in EVCS measures such as traffic filtering, rate limiting, and anomaly detection techniques can be implemented to identify and mitigate abnormal traffic patterns, ensuring uninterrupted and reliable charging services for PEVs [34].
(4) Malware Injections: In an EVCS, the Malware Injection attack involves introducing malicious software into the system, mainly targeting the EVSE units. Since EVSEs are often publicly accessible at charging stations, they can become vulnerable to malware infections. Attackers can exploit these vulnerabilities to inject malware into EVSEs, which can then spread to other units within the network. Once infected, the malware can compromise the security of the entire EVCS ecosystem, including the PEVs, the SCMS, and even the power grids. This attack can result in the theft of sensitive data, such as credit card information and personal details, from unsuspecting users [31]. Implementing robust cybersecurity measures to mitigate Malware Injection attacks, including regular security testing and assessment of EVSEs, is crucial to ensure their integrity and protect the overall EVCS infrastructure from potential malware threats.
(5) Physical Attack: A physical attack in an EVCS refers to any deliberate act of damaging or tampering with the system’s physical components, such as the EVSE or the PEVs. This type of attack can have severe consequences, including personal safety risks and threats to the integrity of the power grid network [35]. For example, an attacker may physically manipulate the charging infrastructure to disrupt the charging process or cause damage to the electrical system. By compromising the synchronized charging activities, the attacker can create disturbances in the grid’s stability and potentially disrupt the overall functionality of the EVCS. Safeguarding against physical attacks in the EVCS requires implementing physical security measures, such as surveillance systems, access controls, and tamper-resistant designs for the charging equipment, to deter and mitigate potential physical threats.

## 4. Cyber Threat/Risks Analysis in EVCS

There is growing concern that EVs may adversely affect power grid reliability due to their unpredictable charging behavior. Some potential attacks on the PEV charging systems have been addressed [36] and discussed in Section 3. The potential negative effects of PEVs integrated components, including a risk to the general public’s safety for nearby residents and those operating nearby vehicles, have been highlighted. The prevalence of cyberattacks against EVs is growing. More charging stations provide more potential targets for cyberattacks on EVs. A hacker could exploit many security holes across brands to gain unauthorized access to user accounts, disrupt charging, or even transform one of the chargers to gain access to an owner’s home network [37].

### 4.1. Charging System Threat Analysis

The battery management system of an EV is vulnerable to attack if an attacker gains access to it through a hacked website or by downloading malware to the EVs systems. Researchers have addressed how attackers might harm EV batteries by altering the charging current and avoiding safeguards [38]. Infrastructure for EVs is inherently vulnerable since IoT devices use various web-based communication and application services [4]. In haste to get their products to market quickly and at a lower cost, operators and manufacturers sometimes compromise on security. The safe functioning of EVs charging is deemed crucial for the security of the new smart grid because of the interconnected nature of the EVs infrastructure and the power grid. The United States Department of Transportation has identified the following as some of the security concerns related to the EV charging ecosystem, and these concerns are discussed in detail [36]:

- The EVs sector needs safer software development practices.
- Communication between EVs and EVCSs is not standardized on a secure communication protocol.
- Insufficient data integrity controls and cybersecurity monitoring systems exist.
- While the physical characteristics of EVCSs vary, many are easily accessible and modifiable.

**Figure 11:**
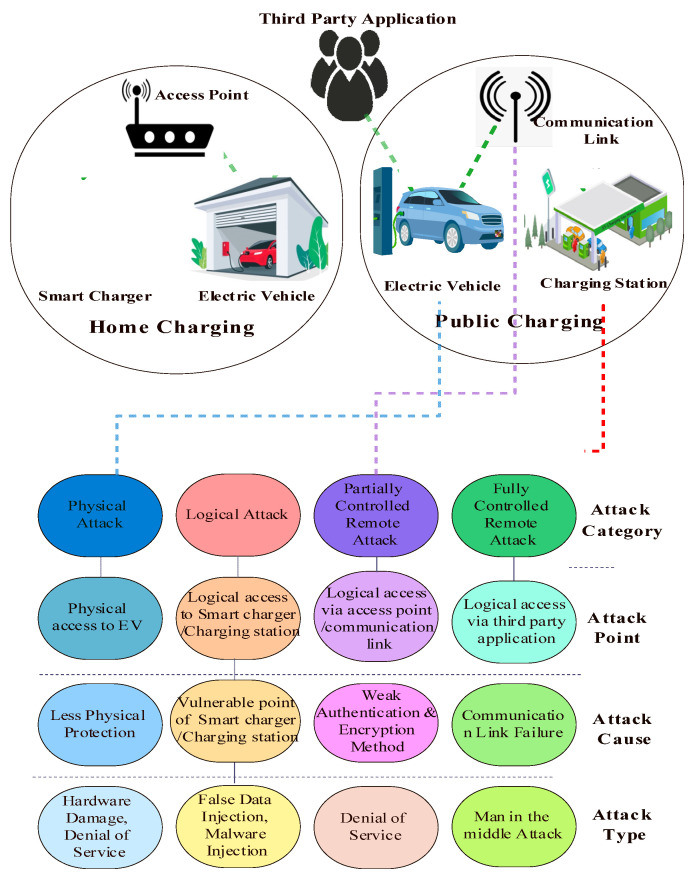
Possible cyberattack on EV charging use cases with vulnerabilities.

A proposal from the National Institute of Standards and Technology (NIST) identifying attacks [39] on the EVs infrastructure is summarized in (Figure 11) as follows:

(1) Physical Attack: Due to its lack of physical protection, EVCSs are vulnerable to attacks that disable the system, steal power, or infect it with malware via accessible USB ports.
(2) Logical Attack: The EVCSs are compromised in such an attack by exploiting a flaw in the firmware, which allows the attacker to acquire logical access to the system. Some suppliers’ firmware upgrades, including those released by Schneider Electric, may be downloaded from the Internet and dissected by attackers to discover security flaws and potential entry points [32]. Kaspersky laboratories could also crack the Charge Point home charger firmware by local attack [40].
(3) Partially controlled Remote Attack: Local Area Networks at charging points may be used by attackers to gain access to the EVCSs. Weak authentication and outdated encryption methods are typical of such systems. Over the charging line, the EVs and EVCSs communicate via a series of protocols, which leaves the system open to attack.
(4) Fully controlled Remote Attack: Users of EVs interact with the EVs management system through an online user interface. Such interaction opens potential vulnerabilities, whether a website or a mobile application.

### 4.2. Charging Session Threat Analysis

Cybersecurity threat analysis involves assessing the security risks associated with EV charging infrastructure, such as the possibility of cyberattacks on charging stations, electric grids, or EV batteries. By analyzing the parameters used in EV charging, cybersecurity experts can identify potential vulnerabilities and design security measures to mitigate these risks.

#### 4.2.1. Experimental Setup

**Figure 12:**
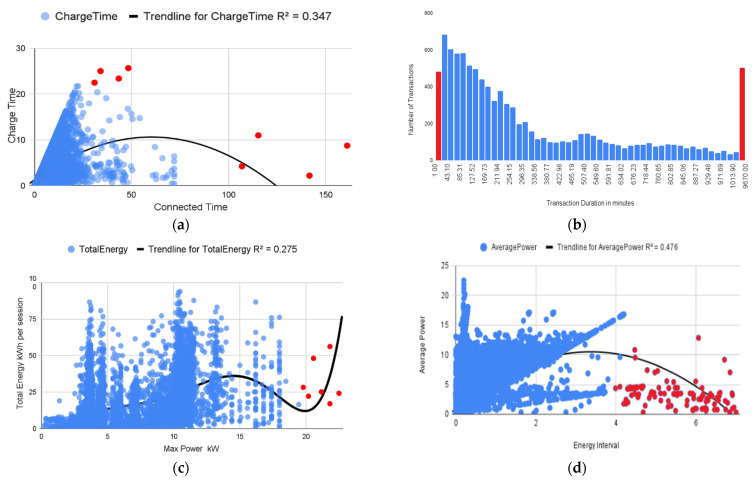
Threat analysis of EV charging sessions: (**a**) Correlation scatter plot for the attributes Connection Time and Charge Time; (**b**) Bar Chart representing Transaction timeframe; (**c**) Correlation scatter plot for the attributes max power and total energy; (**d**) Correlation scatter plot for the attribute’s energy interval and average power.

Transactional data shared by ElaadNL [41], which depict an increase in the use of EVs and their respective rise in usage of Charging Stations, for the year 2019, are used to deploy the charts in Figure 12. The data provided by ElaadNL [41] contain information about various parameters related to charging sessions at different charging stations in the Netherlands. These parameters include total energy consumed, maximum charging power, connected time, charging time, UTC transaction start and stop timestamps, energy interval, and average power. Based on these parameters, statistical methods or machine learning algorithms can detect abnormal behavior in charging sessions. For example, anomaly detection techniques can be applied to identify charging sessions that deviate significantly from the expected behavior of charging sessions for each of these parameters.

Some of the factors that can be considered while detecting abnormal behavior in charging sessions based on these parameters include the following:

(1) Total energy consumed: Charging sessions that consume significantly more or less energy than usual can be considered abnormal.
(2) Maximum charging power: Charging sessions that consume significantly more or less power than usual can be flagged as abnormal.
(3) Connected time: Charging sessions that take an unusually long time or end abruptly can be flagged as abnormal.
(4) Charging time: Charging sessions that take significantly longer or shorter time than usual can be flagged for further investigation.
(5) UTC transaction start and stop timestamps: Charging sessions that start or end at unusual times can be considered abnormal.
(6) Energy interval: Charging sessions with an unusual energy interval between two consecutive energy measurements can be flagged for further investigation.
(7) Average power: Charging sessions that consume significantly more or less power on average than usual can be considered abnormal.

This study employed a methodology for detecting abnormal behavior in EV charging sessions. Transactional data from ElaadNL were collected, encompassing various parameters. Statistical analysis and regression analysis were conducted to identify relationships and correlations among the parameters. Anomaly detection techniques were then applied to identify charging sessions deviating significantly from normal behavior. Visualizations were created to depict the abnormal behavior detected, enabling further analysis and interpretation.

#### 4.2.2. Result and Discussion

Based on the regression analysis performed on the data, the following result shown in Figure 12a–d explains the abnormal behavior detected in various charging sessions.

Charge-Time is the energy transfer duration, while Connected-Time is the difference between the start and end of a transaction. The graph in Figure 12a shows the relationship between Charge-Time and Connected-Time, with an R-squared value of 0.347, indicating that Connected-Time can explain 34.7% of the variation in Charge-Time. Outliers, marked in red, fall outside the 95th percentile range and may warrant further investigation to determine if they are due to a genuine data error or malicious activity. Some records in the dataset exhibit abnormal usage behavior where the Connected Time is 150+ h, but the Charge-Time is less than 10 h. The UTC-Transaction-Start is the start time of a transaction, and the UTC-Transaction-Stop is the stop time of a transaction. The histogram plot Figure 12b displays the Transaction Time in minutes, equivalent to Connected-Time. The chart suggests that transactions typically take between 30 and 1035 min to commence. Transactions that take over 1200 min (20 h) may be due to connection timeouts or suspicious activity. However, for Level 1 or Level 2 infrastructure, it may take 24+ h to charge, whereas DC fast charging usually takes 15 min to 3 h.

The graph in Figure 12c shows the correlation between Max-Power and Total-Energy. The L1 charger’s output is between 1.3 and 2.4 kW, while the L2 charger is between 3 and 19 kW of AC power. Values beyond these ranges for Max-Power could indicate suspicious activity. In addition, Total-Energy consumption per session beyond 75 kWh may indicate heavy battery requirements, which could be a concern. Energy-Interval is the total energy (kWh) transfer between two consecutive meter readings, and Average-Power is the average power in kW between two consecutive meter readings. Figure 12d shows a graph plot of Energy-Interval and Average-Power, where a change in Energy-Interval explains 48% of the change in Average-Power. Some unusual logs where Average-Power is lower than Energy-Interval beyond 4 kWh is marked red and need further investigation.

The threat analysis suggests that the dataset’s charging session data are vulnerable to potential attacks or anomalies. The relationships and patterns between charging session attributes indicate potential issues or suspicious activity. Abnormal usage behavior with extremely long Connected-Time but short Charge-Time, transactions taking longer than expected, and values beyond normal ranges for Max-Power and Total-Energy consumption raise concerns. Logs also indicate anomalies where Average-Power is lower than Energy-Interval beyond a certain threshold. These findings highlight the need for further investigation to determine if these anomalies result from genuine data errors, malicious activities, or potential vulnerabilities in the charging sessions that attackers could exploit.

## 5. Open Research Issues in EVCS

- Cyberattacks on EV Charging Networks.

**Figure 13:**
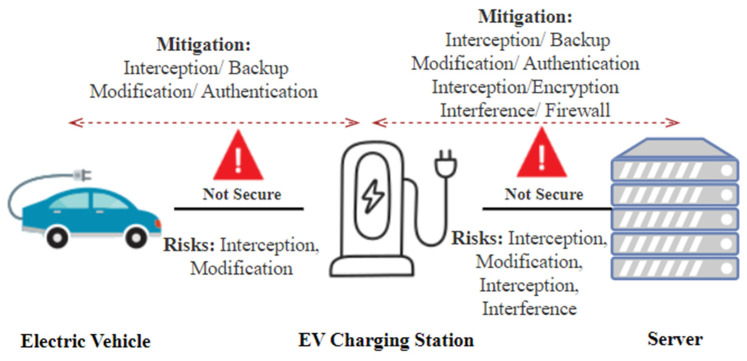
Cyberattacks on EV charging networks.

The issue of cyberattacks on EV charging networks involves abnormal behavior in charging sessions that can impact EVs, charging stations, and EV servers [33], as shown in Figure 13. This issue poses real-time problems such as modification, interruption, interception, and interference [42]. To address this issue, research directions should focus on Authentication, backup, encryption, security monitoring using firewall, user education, and collaboration for establishing standards. These efforts aim to mitigate cyber threats, enhance security, and ensure the reliable and secure operation of EV charging infrastructure.

- Lack of Standardization in EV Charging Networks.

**Figure 14:**
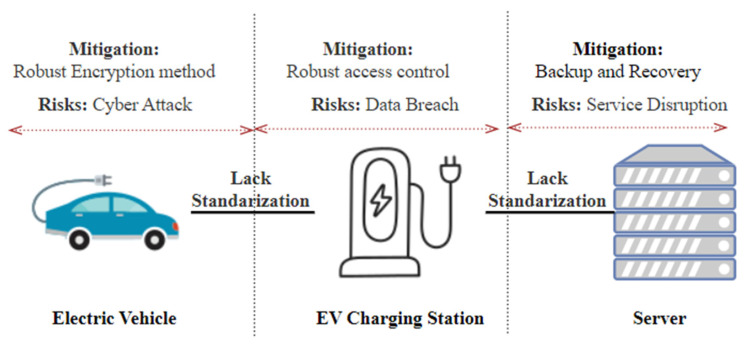
Lack of standardization issue in EV charging network architecture.

The current PEV and EVSE charging infrastructures must meet the best cybersecurity standards already in place [19,43]. The existing infrastructure falls short of established cybersecurity standards and lacks standardized development processes for security software [27], as shown in Figure 14. This issue poses a real-time problem as it leaves the charging infrastructure vulnerable to cyberattacks, potentially leading to unauthorized access, data breaches, and disruptions in charging services. To address this issue, research should focus on developing and implementing robust security frameworks, improving encryption methods, access control, and providing data backup. Additionally, exploring centralized or distributed cloud services can offer potential solutions for improving the security of the charging infrastructure [44,45].

- Insecure End-to-End Communication for EVs Charging

Communication from end to end relies on a trust paradigm still in its formative phases [25]. In the current state, the majority of the Plug-in Electric Vehicle (PEV) and charging infrastructure sectors need more accessible access to cybersecurity testing and assessment [19,26]. This issue creates a real-time problem, raising concerns about the reliability and security of communication channels between EVs, EV charging stations, and servers, as shown in Figure 13. To address this issue, research should focus on developing robust communication protocols, encryption mechanisms, and authentication frameworks to ensure secure and trustworthy end-to-end communication. Additionally, exploring advancements in energy forecasting and communication technologies can enhance the efficiency and reliability of EV charging systems [24].

- Define-Test-Validate Charging Security Guidelines

Existing EV infrastructures have not kept pace with the latest technology advancements [24], and accessible EVSEs still struggle with insufficient physical security standards [46,47,48]. As a result, consumer trust in PEVs has been shaken. To address this issue, it is crucial to establish comprehensive and standardized security solutions for existing EVs, as shown in Figure 14. These guidelines should be tested and validated in real-world scenarios to ensure their effectiveness in mitigating cybersecurity risks and restoring consumer confidence in PEVs. Research efforts should focus on developing robust security frameworks, physical security standards for EVSEs, and conducting practical tests to validate the effectiveness of recommended security measures [49,50].

- AI-enabled EV Charging

Considering the capability of AI in detecting or predicting future events, the application of AI for addressing charging related cyber risks is another future direction of EV cyber research themes [51,52]. Some existing research has shown the potential of using AI in EV research [53]. However, its potential impact on EV cyber research is yet to be explored with specific use cases in securing EV charging networks and detecting risks in EV charging networks.

## 6. Conclusions

This paper critically analyzes EV charging architecture, vulnerable points in the charging network, cyberattacks, and communication protocols used by current EVs. In addition to a literature review on the most commonly occurring cyber-physical threats against charging infrastructure and their implications, it also provided threat analysis on charging session data to prove that charging sessions have abnormalities leading to cyber threats. Also covered are some open issues and research gaps in the currently available SCMS. SCMS aims to maximize the rapid charging of many PEVs and offer various grid services. As a result, further work is required in securing SCMSs concerning their communication protocols. The deployment of SCMS involves physical components vulnerable to several cyber threats. Therefore, cybersecurity for SCMS requires the implementation of appropriate detection, identification, protection, and mitigation methods. In addition, present commercially available SCMS face unique cybersecurity-related dangers. Therefore, further research and a greater understanding of rules and security are required. Future research can explore the application of machine learning and artificial intelligence techniques in enhancing the security of EV charging architecture and SCMS. This can involve developing intelligent algorithms for anomaly detection, predictive analysis of cyber threats, and adaptive security measures. Additionally, investigating the use of AI-based methods for securing communication protocols and addressing vulnerabilities in SCMS can be a valuable area of study.

## Figures and Tables

**Table 1:** Summary of pros and cons of EVCS use cases.

Pros of Home Charging Infrastructure	Cons of Home Charging Infrastructure	Pros of Public Charging Infrastructure	Cons of Public Charging Infrastructure
More convenience, more savings, increased home value, less wear and tear, less susceptible to attack	Longer charging time, higher upfront Cost	No investment, more economical	Battery damage, longer waiting time, inconvenience in searching, more susceptible to attack

## Data Availability

Research data will be available on individual requests to the corresponding author considering collaboration possibilities with the researcher or research team and with restrictions that the data will be used only for further research in the related literature progress.
